# Current status of the development of dengue vaccines

**DOI:** 10.1016/j.jvacx.2024.100604

**Published:** 2024-12-17

**Authors:** Michelle Felicia Lee, Chiau Ming Long, Chit Laa Poh

**Affiliations:** a Department of Biological Sciences, School of Medical and Life Sciences, Sunway University, Selangor 47500, Malaysia; b Department of Medical Sciences, School of Medical and Life Sciences, Sunway University, Selangor 47500, Malaysia; c ALPS Global Holding Berhad, The ICON, East Wing Tower, No. 1, Jalan 1/68F, Off Jalan Tun Razak, Kuala Lumpur 50400, Malaysia

**Keywords:** Flavivirus, Dengue virus, Vaccine, Vaccine development, Live-attenuated vaccine

## Abstract

Dengue fever is caused by the mosquito-borne dengue virus (DENV), which is endemic in more than 100 countries. Annually, there are approximately 390 million dengue cases, with a small subset manifesting into severe illnesses, such as dengue haemorrhagic fever or dengue shock syndrome. Current treatment options for dengue infections remain supportive management due to the lack of an effective vaccine and clinically approved antiviral. Although the CYD-TDV (Dengvaxia®) vaccine with an overall vaccine efficacy of 60 % has been licensed for clinical use since 2015, it poses an elevated risk of severe dengue infections especially in dengue-naïve children below 9 years of age. The newly approved Qdenga vaccine was able to achieve an overall vaccine efficacy of 80 % after 12 months, but it was not able to provide a protective effect against DENV-3 in dengue naïve individuals. The Butantan-DV vaccine candidate is still undergoing phase 3 clinical trials for safety and efficacy evaluations in humans. Apart from live-attenuated vaccines, various other vaccine types are also currently being studied in preclinical and clinical studies. This review discusses the current status of dengue vaccine development.

## Highlights

• The search for a more efficient tetravalent dengue vaccine candidate is still on-going.
• There are currently two licensed live-attenuated dengue vaccines, CYD-TDV (Dengvaxia®) and Qdenga.
• However, they are not able to provide long-lasting, balanced immune protection against all four DENV serotypes.
• Various other types of vaccines are also currently being studied in preclinical and clinical studies.

## 1 Introduction

Dengue virus (DENV) is widespread in tropical and sub-tropical regions, and its predominance is influenced by various factors such as humidity, travel, and urbanization. It is endemic in over 100 countries especially in Southeast Asia, Africa, the Americas, the Mediterranean, and the Western Pacific, with an estimated 390 million cases yearly. The Americas, Western Pacific, and Southeast Asia were the most severely afflicted, with 70 % of the worldwide dengue disease burden being reported in Asia [1]. Several large dengue outbreaks have occurred in countries such as Nepal, Bangladesh, China, Myanmar, and Laos in the past few years [2,3]. A prediction model by Messina et al. (2019) estimated that the number of dengue-infected individuals would increase by 2.25 billion from 2015 to 2080 [4]. The rise in temperatures due to climate change is a growing concern as dengue will worsen in endemic regions due to increased mosquito survival, biting rate, and reproduction, which could lead to longer dengue transmission seasons and an increased number of dengue cases. Additionally, the rise in temperatures could also lead to increased dengue transmission and dissemination in low-risk or dengue-free regions such as Australia, North America, and Europe. Due to the growing concern regarding dengue, the World Health Organization (WHO) has listed dengue as one of the top 10 most significant global health threats [5]. The WHO reported a total of 7.6 million dengue cases in 2024 alone, which includes 3.4 million active cases, 16,000 severe dengue cases, and an estimated total of over 3000 deaths. This increase is more prominent in the Americas, as the number of dengue cases exceeded 7 million as of April 2024, which far surpassed the total of 4.6 million dengue cases in 2023 [6].

DENV is classified under the genus *Flavivirus* of the *Flaviviridae* family [7]. It is primarily transmitted via female mosquito vectors such as *Aedes aegypti* and *Aedes albopictus* [8]. DENV consists of four different serotypes, DENV-1 to -4, which shared 65–70 % amino acid sequence identity, and they can be further subdivided into several phylogenetic groups with unique genotypes [9]. All four DENV serotypes are known to co-circulate worldwide. The WHO reported that DENV-3 and DENV-4 have a higher frequency of occurrence in 2023 [6]. Dengue infection can be asymptomatic or symptomatic depending on the infected individual. The common clinical symptoms in a dengue-infected individual include headache, fever, nausea, vomiting, fatigue, arthralgia, muscle pain, chills, retro-orbital pain, lymphitis, urticaria, and leukocytopenia [10]. Severe dengue infection can escalate to dengue hemorrhagic fever, which is characterized by increased vascular permeability and hemostatic irregularities, as well as dengue shock syndrome or hypovolemic shock [11]. Infection with a single serotype generates life-long immune protection against that specific serotype and short-lived immune protection against the other three heterologous serotypes [12]. However, following a primary infection of a particular DENV serotype, a secondary infection with any of the other three heterologous serotypes could lead to severe dengue infection. The phenomenon known as antibody-dependent enhancement (ADE) or original antigenic sin is a leading mechanistic explanation for severe disease during secondary DENV infections [13,14].

The dengue virion is a spherical, enveloped particle consisting of a lipid bilayer with an icosahedral outer shell and a nucleocapsid core containing the RNA genome. It has a positive-sense single-stranded RNA genome which is approximately 11 kilobases in size with two untranslated regions (UTRs) at both ends of the genome [15]. The 5’-UTR is made up of 95–135 nucleotides and a type I cap-like mRNA, primarily functioning to control gene expression via regulation of translation. On the other hand, the 3’-UTR is made up of 114–650 nucleotides, lacks a poly(A) tail, and contains a conserved stem-loop secondary structure which plays a vital role in viral replication [[16], [17], [18]]. The RNA genome encodes for a single polyprotein, which is further cleaved into three structural proteins, capsid (C), precursor membrane (prM) or membrane (M), envelope (E) proteins, and seven non-structural (NS) proteins, NS1, NS2A, NS2B, NS3, NS4A, NS4B, and NS5 proteins [19]. The structural proteins form the DENV viral particle with the E protein functioning for host cell entry and identification via the fusion process between the viral envelope and host cell membrane [20]. The NS proteins play vital roles in various stages of the DENV life cycle including viral replication, polyprotein cleavage, virion maturation and assembly [[21], [22], [23], [24]].

The existing vector control strategies are insufficient to prevent human infection and rising rates of dengue infection. The development of a safe and effective dengue vaccine is vital to reduce the global dengue disease burden [25]. Currently, there are two licensed live-attenuated dengue vaccines, Dengvaxia® and Qdenga, which have been approved for use in several dengue endemic countries [[26], [27], [28], [29], [30]]. Another live-attenuated dengue vaccine, Butantan-DV is currently undergoing phase 3 clinical trials [31,32]. Apart from that, there are various other vaccine candidates, which are currently undergoing preclinical and early clinical studies. This review discusses the current status of dengue vaccine development.

## 2 Antibody-dependent enhancement (ADE) in dengue infections

**Fig. 1:**
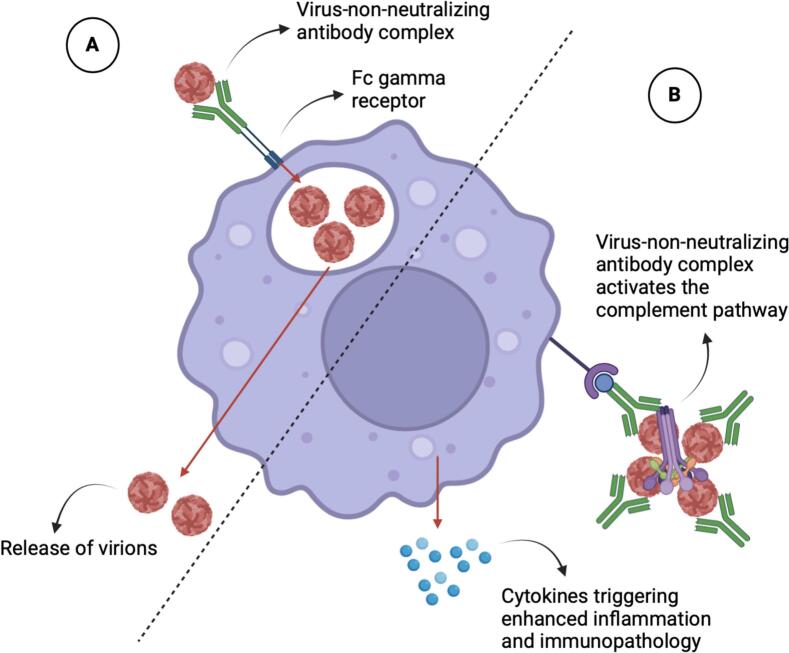
Schematic illustration of the types of ADE mechanisms. (A) Extrinsic ADE involves an increased uptake of viruses into phagocytic cells via the Fc gamma receptors, leading to increased viral replication. (B) Intrinsic ADE involves complement activation by the formation of virus-non-neutralizing antibody complexes, which caused virus-tolerant states, increased inflammation, and immunopathology. The original figure was created using Biorender software.

ADE in viral infections is characterized by two mechanisms, extrinsic ADE and intrinsic ADE. Extrinsic ADE involves an increased uptake of viruses into phagocytic cells via the Fc gamma receptors, leading to increased viral replication. Intrinsic ADE involves complement activation by the formation of virus-non-neutralizing antibody complexes, which caused virus-tolerant states, increased inflammation, and immunopathology [33]. Fig. 1 provides a brief overview of the two types of ADE mechanisms. The key receptor in these ADE mechanisms is the Fc gamma receptor, which are present on the surface of immune cells to facilitate the viral infection. It also functions as a receptor for the Fc portion of immunoglobulin G (IgG) present on various cells including B cells, mast cells, macrophages, and dendritic cells [33].

Studies have found that primary dengue infections induce strong neutralizing antibody responses, thus conferring long-term immune protection against the primary infecting DENV serotype, but only short-lived immune protection against the other DENV serotypes. The duration of the short-lived cross protection against the other heterologous DENV serotypes has been hypothesized to last for 1–2 weeks, 8 weeks, a year or longer [[34], [35], [36]]. These neutralizing antibodies would eventually be replaced by non-neutralizing cross-reactive antibodies [37]. An extrinsic ADE mechanism occurs when these non-neutralizing antibodies bind to viruses without clearing the infection. These non-neutralizing antibodies bind to the surface glycoproteins of the viruses, rendering them more prone for engulfment by phagocytic cells through the Fc gamma receptor [38,39]. Upon ligation of the immunocomplex with the Fc gamma receptor, the complex then enters the endosome of the cell. The complex is then released inside the cell once the affinity of Fc gamma receptor to IgG is low. The dissociation of the immunocomplex from the Fc gamma receptor switches the antiviral innate immune mechanism to an immune-suppressive mechanism [33].

Apart from extrinsic ADE, intrinsic ADE also contributes to enhancing dengue viral replication. It was previously reported that the upregulation of the biosynthesis of interleukin-10 via intrinsic ADE plays a significant role in the suppression of host-mediated innate and adaptive immune responses during a dengue infection. Other than increasing viral replication, dengue-induced ADE was also found to induce a Th2-type immune response via increased production of interleukin-6 and interleukin-10. This results in overexpression of the suppressor of cytokine signaling 3 gene (SOCS3), which leads to inhibition of the Janus kinase-signal transducer and activator of transcription (JAK-STAT) signaling pathway and interferon gamma production. Additionally, this also leads to inhibition of the synthesis of nitric oxide, which increases dengue viral RNA synthesis [39,40].

## 3 Original antigenic sin

**Fig. 2:**
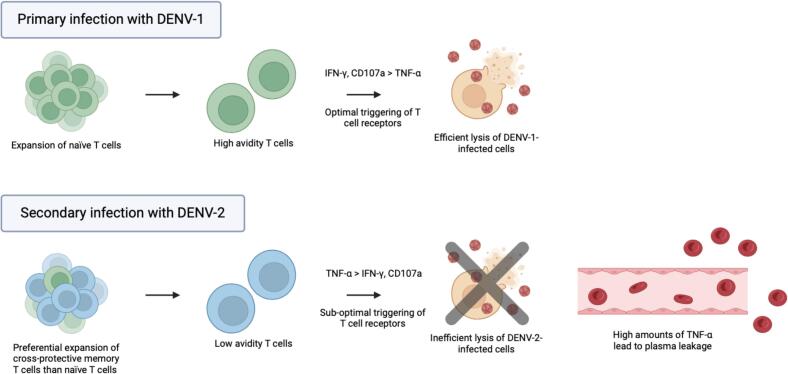
Potential impact of original antigenic sin of T cells on DENV-specific T cell responses during a secondary DENV infection. During a primary infection (e.g. with DENV-1), there is an expansion of naïve T cells with high avidity for serotype-specific DENV-1 epitopes. These T cells undergo optimal T cell receptor triggering, which lead to the production of IFN-γ and CD107a, resulting in the efficient lysis of DENV-infected cells. In the event of a secondary infection with a heterologous serotype (e.g. DENV-2), cross-reactive memory T cells specific for the primary infecting virus, DENV-1, dominate the response due to their increased numbers and their lower activation threshold as compared to naïve T cells. Some of these cells may have a lower avidity for DENV 2 antigens, which result in suboptimal T cell receptor triggering and subsequent production of high levels of TNF-α and low levels of CD107a and IFN-γ, leading to an inefficient lysis of DENV-infected cells. Secretion of high amounts of TNF-α may contribute to the cytokine storm and plasma leakage. The original figure was created using Biorender software.

The phenomenon known as original antigenic sin arises from cross-reactive memory T cells generated during a primary dengue infection. This phenomenon refers to the immunological memory of the primary infecting DENV being favored over the secondary infecting DENV encountered much later, resulting in cross-reactive memory T cells specific for the primary infecting DENV, dominating the cellular immune responses during a secondary dengue infection. Although large numbers of T cells are rapidly activated during the secondary dengue infection, they are less efficient in the lysis of DENV-infected cells due to sub-optimal triggering of T cell receptors. Consequently, this leads to poor DENV clearance in the host and alteration of the pattern of cytokine production. This enhances the production of pro-inflammatory cytokines such as tumor necrosis factor alpha (TNF-α) and reduces the production of antiviral cytokines such as interferon-gamma (IFN-γ), which may contribute to the development of severe dengue infection (Fig. 2) [41,42].

## 4 Dengue vaccines

**Fig. 3:**
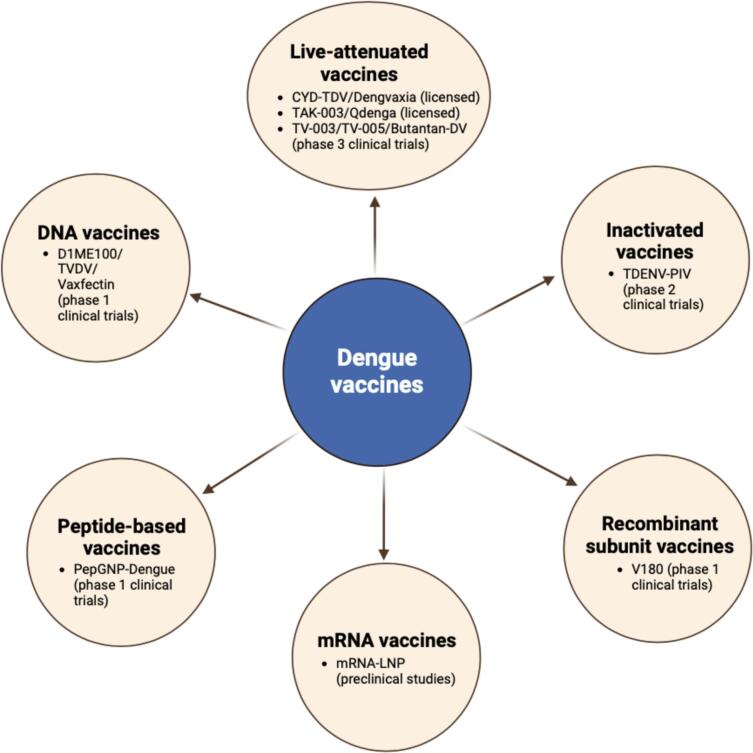
Types of dengue vaccines. The original figure was created using Biorender software.

The development of dengue vaccines is an on-going effort due to the lack of a vaccine with balanced protection against all four DENV serotypes. Various types of dengue vaccines, which have been developed include live-attenuated vaccines, inactivated vaccines, DNA vaccines, recombinant subunit vaccines, peptide-based vaccines, and mRNA vaccines (Fig. 3).

### 4.1 Live-attenuated vaccines

Live-attenuated vaccines utilize live pathogens, with limited virulence in immunization. Live virus vaccines could induce both humoral and cellular immune responses against DENV structural and NS proteins. However, vaccines derived from antigenic chimeric viruses could only induce a limited response to NS proteins [43]. Apart from dengue, live-attenuated vaccines have also been developed for yellow fever virus (YFV) and Japanese encephalitis virus (JEV). These are the YF-17D and JEV SA14–14-2 vaccines which provided over 90 % long-term efficacy with the administration of a single dose [44,45]. Since dengue infections induce life-long immune protection against the specific serotype, live-attenuated vaccines are expected to mimic natural dengue infection to stimulate the cellular and humoral immune responses to provide long-term immune protection. The CYD-TDV/Dengvaxia® vaccine produced by Sanofi Pasteur, TAK-003/Qdenga vaccine produced by Takeda, and TV-003/TV-005/Butantan-DV vaccine produced by the Butantan Institute utilizes recombinant live-attenuated viruses (Table 1) [46].

**Table 1:** Licensed dengue vaccines and dengue vaccine candidates currently in preclinical/clinical trials.

Type of vaccine	Vaccine name	Status	Description	Limitations	References
Live-attenuated vaccines	CYD-TDV/ Dengvaxia®	Licensed	• Consists of the YF-17D vaccine strain as the backbone and the prM and E genes of YF-17D were replaced with those of the four DENV serotypes. • Preclinical studies: o Protected cynomolgus macaques challenged with DENV from viremia and RNAemia post-DENV challenge. o Predominant DENV-1 and DENV-4 neutralizing antibody response following the vaccination. • Phase 1 and 2 trials: o Provided data on the safety and immunogenicity of the vaccine formulations and immunization schedules. o Final vaccine formulation and schedule for CYD-TDV: 5 log10 CCID50 of each live-attenuated DENV-1 to -4 given in three doses with 6 months apart. • After licensing: o The overall vaccine efficacy in children aged between 2 and 17 years old was determined to be 60 %. o Protection against DENV-3 and DENV-4 were 75 % and 77 %, respectively, but protection against DENV-1 and DENV-2 were much lower at 51 % and 34 %, respectively.	• Increased hospitalization risk among vaccinees 3 years following the vaccination. • Low vaccine efficacy against DENV-2. • High risk of dengue-related hospitalizations 3 years after vaccination for children below 9 years of age. • Enhanced risk of hospitalization for seronegative vaccinees who were infected with DENV-2 much later.	[[26], [27], [28],[46], [47], [48], [49], [50], [51], [52], [53], [54]]
	TAK-003/Qdenga	Licensed	• Consists of the live-attenuated DENV-2 PDK-53 strain (TDV-2) as the backbone and the DENV-2 prM and E genes were substituted with those from DENV-116007 (TDV-1), DENV-316562 (TDV-3), and DENV-41036 (TDV-4). • Preclinical studies: o Conferred protection against all four DENV serotypes in non-human primates, though it induced lower antibody responses against DENV-4. • Phase 1 trials: o One vaccine dose was sufficient to generate the necessary amount of neutralizing antibodies to inhibit DENV-2 replication. o No serious adverse events recorded and most of the recipients only experienced erythema and pain at the injection site. • Phase 2 trials: o No serious adverse events recorded and most of the recipients only experienced erythema and pain at the injection site. o Two dose schedules were more effective than the one dose schedule in increasing seropositivity rates in dengue-naïve individuals. • Phase 3 trials: o Overall vaccine efficacy for the timeframe between 12 and 18 months was determined to be 73.3 %. o In the second year after vaccination, the vaccine efficacy dropped to 56.2 % and it was suggested that this could be due to a change in the predominant DENV serotype. • After licensing: o Well-tolerated and not linked to any serious adverse events. o In the first year post-vaccination, a vaccine efficacy of 80 % was observed against virologically confirmed dengue fever (VCD). o Its efficacy against VCD was the highest against DENV-2 at 98 % and the lowest against DENV-3 at 63 %. o At 4–5 years post-vaccination, the cumulative vaccine efficacy in preventing VCD dropped to 59 %.	• Not able to provide protective effect against VCD caused by DENV-3 in dengue naïve individuals. • No vaccine efficacy studies have been carried out in individuals >16 years old.	[29,30,46,[55], [56], [57], [58], [59], [60], [61], [62], [63], [64]]
	TV-003/TV-005/Butantan-DV	Phase 3 clinical trials	• Developed via the creation of a series of attenuated DENV with introductions of nucleotide deletions in the 3’-UTR and mutations in the NS proteins. • Phase 1 trials: o Among the four vaccine formulations, TV-003 induced the most balanced antibody responses against all four DENV serotypes. o Administration of a single dose of TV-005 elicited a tetravalent response in 90 % of the vaccinees, compared to 76 % of the TV-003 vaccinees after 3 months of vaccination. • Phase 2 trials: o Most of the vaccinees (88 %–92 %) only experienced a mild rash. o The seroconversion rates at 91 days after administration of the first dose were observed to be 94 %, 82 %, 82 %, and 88 % for DENV-1 to -4, respectively. • Phase 3 trials: o The overall 2-year vaccine efficacy was determined to be 79.6 %. o The vaccine efficacies against DENV-1 and DENV-2 were determined to be 89.5 % and 69.6 %, respectively.	• No data for vaccine efficacy of DENV-3 and DENV-4 was recorded.	[31,32,46,[65], [66], [67], [68]]
Inactivated vaccines	TDENV-PIV	Phase 2 clinical trials	• Consists of all four DENV serotypes which were chemically inactivated using formalin to inhibit DENV infectivity, while maintaining their structures and antigenicity. • Phase 1 trials: o Demonstrated a good safety profile with mild adverse events such as minimal injection site reactions. o Administrations with adjuvants AS03B and AS01E were found to induce the highest mean antibody titers. • Phase 2 trials: o Participants administered with TDENV-PIV vaccine with AS03B adjuvant reported adverse events within 7 days after administration.	• Lower immunogenicity than live-attenuated vaccines.	[[69], [70], [71], [72], [73]]
DNA vaccines	D1ME100/ TVDV/Vaxfectin	Phase 1 clinical trials	• Consists of four plasmids carrying genes encoding for the E and prM proteins from each DENV serotype. • Phase 1 trials: o Was proven to be safe and well-tolerated in the initial immunization phase.	• Weak immunogenicity was observed in participants who received high-dose immunization. • No neutralizing antibody response was detected in participants who received low-dose immunization. • High DENV-specific IFN T-cell response was generated in approximately 79 % of the high dosage vaccinees.	[[74], [75], [76], [77]]
Recombinant subunit vaccines	V180	Phase 1 clinical trials	• Recombinant DENV E vaccine. • Preclinical studies: o V180 and ISCOMATRIX adjuvant were administered in mice and monkeys to evaluate their efficacy in providing protection against viremia and generating strong neutralizing antibodies against all four DENV serotypes. • Phase 1 trials: o All the vaccine formulations were well-tolerated by the study participants and strong immunity was observed among the study participants. o The vaccine formulation with ISCOMATRIX adjuvants was found to be more immunogenic than vaccine formulations with aluminum adjuvants and without adjuvants.	• Concerns such as protein misfolding and exposure to endotoxins.	[[78], [79], [80], [81], [82], [83]]
Peptide-based vaccines	PepGNP-Dengue	Phase 1 clinical trials	• A gold nanoparticle-based, multivalent, synthetic peptide dengue vaccine candidate. • Designed to provide CD8^+^ T-cell immunity, without eliciting anti-DENV antibodies. • Phase 1 trials: o Only mild adverse events such as pain at the injection site and transient discoloration of the skin were reported. o In the low dose PepGNP-Dengue group, significant increases in the CD8^+^ T cells and dengue dextramer+ memory cell subsets were observed, but not in the high dose PepGNP-Dengue or vehicle-GNP groups.	• Did not elicit anti-DENV antibodies as expected.	[84]
mRNA vaccines	mRNA-LNP	Preclinical studies	• Developed by transcribing the DENV-1 prM and E proteins into a modified mRNA using pseudo uridine, and then packing it into lipid nanoparticles (LNP). • Preclinical studies: o Exerted DENV-1-specific immune response in the AG129 mice. o The formulation consisting of prME, E80, and NS1 proteins of DENV-2 was able to induce the production of neutralizing antibodies against DENV-2 and generate T cell immune responses in the BALB/c mice.	• The mRNA vaccine technology is relatively new and long term adverse effects in humans are unknown.	[85,86]

#### 4.1.1 CYD-TDV/ Dengvaxia®vaccine

**Fig. 4:**
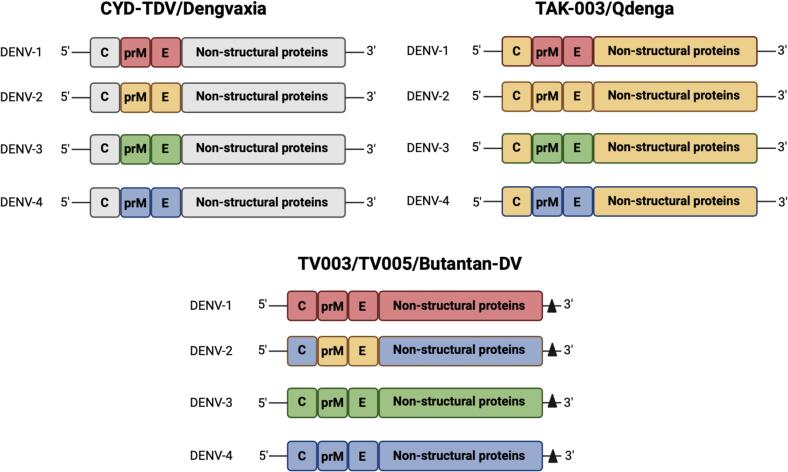
Schematic representation of the developmental strategies of the three live-attenuated dengue vaccines. The backbone of the CYD-TDV/Dengvaxia vaccine consists of the YF-17D vaccine strain and the prM and E genes of YF-17D were replaced with those of the four DENV serotypes. The TAK-003/Qdenga vaccine consists of the live-attenuated DENV-2 PDK-53 strain (TDV-2) as the backbone and the DENV-2 prM and E genes are substituted with those from DENV-116007 (TDV-1), DENV-316562 (TDV-3), and DENV-41036 (TDV-4). The TV003/TV005/Butantan-DV vaccine is developed via the creation of a series of attenuated DENV with introductions of 30 nucleotide deletions in the 3’-UTR and mutations in the NS proteins. Grey = YF-17D virus, red = DENV-1, yellow = DENV-2, green = DENV-3, blue = DENV-4, the black triangle represents the mutations at the 3’-UTR. The figure was created using Biorender software. (For interpretation of the references to colour in this figure legend, the reader is referred to the web version of this article.)

The tetravalent live-attenuated CYD-TDV (Dengvaxia®) vaccine developed by Sanofi Pasteur is the first licensed dengue vaccine for clinical application in individuals aged 9–44 years [87]. The backbone consists of the YF-17D vaccine strain and the prM and E genes of YF-17D were replaced with those of the four DENV serotypes (Fig. 4**)**. The CYD-TDV overall vaccine efficacy in children aged between 2 and 17 years old was determine to be 60 %/ Its protection against DENV-3 and DENV-4 were determined to be 75 % and 77 %, respectively, but protection against DENV-1 and DENV-2 were much lower at 51 % and 34 %, respectively [[26], [27], [28]]. It was reported that there was an increased hospitalization risk among vaccinees 3 years following the vaccination [47,48]. The low overall vaccine efficacy could be due to the lack of DENV NS proteins in the vaccine formulation. A study reported that majority of the conserved epitopes across all four DENV serotypes were located in the NS proteins [88]. Therefore, the lack of CD8^+^ T cell immune responses and neutralizing antibodies against DENV NS proteins potentially contributed to the reduced immune protection conferred by the CYD-TDV vaccine [89]. The low vaccine efficacy against DENV-2 could also be due to lower expression or lower immunogenicity of the DENV-2 prM and E genes in the vaccine formulation [46].

The 2016 CYD-TDV national immunization campaign in the Philippines and Brazil was discontinued in 2017 due to the deaths of 14 children following vaccinations [90]. A report revealed that there was a higher risk of dengue-related hospitalizations 3 years after CYD-TDV vaccination for children below 9 years of age [49]. Another study re-analyzed the data from previous clinical trials and concluded that seronegative children aged between 9 and 16 years old who were vaccinated with CYD-TDV exhibited a high risk for dengue-related hospitalization and severe dengue. It was hypothesized that this increase in risk began approximately 18 months following the administration of the last vaccine dose [48]. Analyses of the data from clinical trials revealed that the CYD-TDV vaccine provided the greatest immune protection against DENV-4 and the least immune protection against DENV-2. Additionally, the CYD-TDV vaccine also enhanced the risk of hospitalization for seronegative vaccinees who were infected with DENV-2 much later [50]. Therefore, the Advisory Committee on Immunization Practices (ACIP) recommended CYD-TDV vaccine to be used for dengue prevention only in children aged between 9 and 16 years old living in dengue endemic regions with pre-confirmed dengue infection [46]. Clinical phase 3 data supported vaccinating seropositives as risk enhancement was greater in seronegatives [50].

Preclinical studies of the CYD-TDV vaccine were carried out in non-human primates such as the cynomolgus macaques (*Macaca fascicularis*). A single dose of the CYD-TDV vaccine was administered subcutaneously to the cynomolgus macaques and tetravalent neutralizing antibody seroconversion was observed. After 6 months, the cynomolgus macaques were challenged with DENV, and it was observed that 22 of the 24 monkeys were protected from viremia [52]. In another study involving cynomolgus macaques, the tetravalent vaccine containing equal concentrations (5 logs) of each DENV viral antigen was administered. DENV-1 and DENV-4 demonstrated a predominant neutralizing antibody response following the vaccination [53]. Another modified formulation with reduced DENV-4 component (5 logs of DENV-1 to -3, 3 logs of DENV-4) demonstrated a DENV-1 predominant neutralizing antibody response following the vaccination [91]. A subsequent study administered two doses of CYD-TDV to cynomolgus macaques, followed by intravenous challenge with 10^7^ CCID50 of DENV after 8 months post-vaccination. It was found that 6 of the 18 monkeys were protected from RNAemia (presence of RNA in the plasma) post-DENV-2 challenge. On the other hand, all the monkeys were protected from RNAemia post-DENV-4 challenge. For the rest, the RNAemia levels were 1–3 logs lower when compared to the controls [54].

The phase 1 trial using the monovalent DENV-2 formulation (CYD01) was performed in the United States [92]. The subsequent phase 1 trials using other formulations such as CYD02 and CYD04 evaluated the safety of the CYD-TDV vaccine in adults from non-endemic areas in the United States. Formulations such as CYD05 and CYD06 evaluated the safety of the CYD-TDV vaccine in adults and children in endemic and non-endemic areas in the Philippines and Mexico, respectively [[93], [94], [95]]. These studies were conducted in non-endemic areas first to collect data from dengue-naïve individuals prior to vaccination. These phase 1 and subsequent phase 2 studies (CYD10, CYD11, and CYD12 formulations) provided data on the safety and immunogenicity of the vaccine formulations and immunization schedules. Following these studies, the final vaccine formulation and schedule was selected: 5 log10 CCID50 of each live-attenuated DENV-1 to -4 given in three doses with 6 months apart. Additional phase 2 trials were also performed in multiple endemic and non-endemic countries in Australia, Asia and the United States to address various concerns regarding dosage, schedule, priming by other flavivirus vaccines, and safety of co-administration with other vaccines. A total of four phase 3 studies (CYD17, CYD29, CYD32, and CYD33 formulations) were subsequently performed without an assessment for an efficacy clinical endpoint [51].

#### 4.1.2 TAK-003/Qdenga vaccine

The tetravalent live-attenuated Takeda dengue vaccine (TAK-003) consists of the live-attenuated DENV-2 PDK-53 strain (TDV-2) as the backbone and the DENV-2 prM and E genes were substituted with those from DENV-116007 (TDV-1), DENV-316562 (TDV-3), and DENV-41036 (TDV-4) (Fig. 4) [55]. The serial passaging of the wild-type DENV-216681 strain in primary dog kidney cells gave rise to the DENV-2 PDK-53 strain. The attenuated DENV-2 PDK-53 strain demonstrated different features from its parental strain such as attenuated neurovirulence, greater genetic diversity, temperature sensitivity, and less effective replication. The NS1–53 Gly-to-Asp, 5’-NCR-57C-to-T, and NS3–250 Glu-to-Val mutations contributed to these features [96,97].

The DENV-2 PDK-53 strain was studied as a single or monovalent component of the multivalent live-attenuated dengue vaccine candidates in Thailand and the United States. It was shown that the DENV-2 PDK-53 strain was highly immunogenic, safe, well-tolerated, and could generate long-lasting immune protection against DENV-2 [[98], [99], [100]]. However, reversed mutations posed a risk in live-attenuated vaccines. An *in vitro* study suggested that the simultaneous reverse mutation of two attenuating mutations at NS1–53 and 5’-NCR-57 could cause the reversion of the DENV-2 PDK-53 strain back to the virulent wild-type DENV-216681 strain [96]. At present, these back-mutations were yet to be observed in any of the dengue vaccine candidates [101].

Preclinical evaluations revealed that the serotype-specific neutralizing antibody titer elicited by the tetravalent live-attenuated vaccine formulation was lower when compared to those elicited by monovalent live-attenuated vaccine. Among the four DENV components of TAK-003, TDV-3 and TDV-4 were found to be less reactogenic and immunogenic than TDV-1 and TDV-2, thereby suggesting potential viral interference among the four DENV serotypes [55]. Several vaccine formulations with different ratios of the four DENV components were prepared and evaluated to overcome this issue. It was observed that vaccine formulations with equal amounts of the four DENV components (10^3^ or 10^5^ plaque-forming units, PFU) failed to induce an adequate amount of neutralizing antibody titer against DENV-4 after two rounds of immunization in AG129 mice and cynomolgus monkeys [102,103]. However, vaccine formulations consisting of 10^3^ PFU of TDV-1 and TDV-2 as well as 10^5^ PFU of TDV-3 and TDV-4 demonstrated a substantial increase in neutralizing antibody titers against DENV-3 and DENV-4. This further confirmed that the differences in replication or immunogenicity between all four DENV serotypes could lead to serotype interference [91]. A previous study found that regardless of the composition of vaccine formulation, the absence of post-challenge viremia suggested that the neutralizing antibodies elicited could neutralize or reduce DENV replication efficiency. Furthermore, there were no reported adverse side effects in all immunized cynomolgus monkeys [103]. In summary, the preclinical data on TAK-003 indicated that it could confer protection against all four DENV serotypes in non-human primates, though it induced lower antibody responses against DENV-4 [46].

A placebo-controlled phase 1 clinical trial assessed a low-dose and a high-dose formulation (NCT01224639, NCT01110551). The low-dose formulation consisted of 8 × 10^3^ PFU of TDV-1, 5 × 10^3^ PFU of TDV-2, 1 × 10^4^ PFU of TDV-3, and 2 × 10^5^ PFU of TDV-4, whereas the high-dose formulation consisted of 2 × 10^4^ PFU of TDV-1, 5 × 10^4^ PFU of TDV-2, 1 × 10^5^ PFU of TDV-3, and 3 × 10^5^ PFU of TDV-4. Two doses of each formulation with 90 days gap were administered via subcutaneous or intradermal injections to dengue-naïve healthy adults. There were no observed clinically meaningful differences in adverse events between the vaccine and placebo groups for both formulations. It was reported that most of the recipients experienced erythema and pain at the injection site. However, the high-dose formulation which was administered intradermally resulted in 77.8 % seroconversion to DENV-1 to -4 after the two doses. Additionally, the geometric mean titers (GMT) of neutralizing antibodies against DENV-1 and DENV-2 were also increased significantly. Overall, the GMT was the highest for DENV-2, followed by DENV-3, DENV-1, and DENV-4. It was also observed that regardless of the routes of administration and formulations, the second administered dose did not significantly increase the antibody titer against DENV-2, but slightly increased the antibody titers against DENV-1, DENV-3, and DENV-4. This suggested that one vaccine dose was sufficient to generate the necessary amount of neutralizing antibodies to inhibit DENV-2 replication [59,60].

In the phase 2 clinical trial, apart from dengue-naïve individuals, the high-dose formulation was also evaluated in individuals that were seropositive for at least one DENV serotype (NCT01511250). Similar to the data from phase 1 clinical trials, there were no serious adverse events recorded and most of the recipients only experienced erythema and pain at the injection site. This implied that the pre-existing anti-DENV antibodies did not increase reactogenicity or the amount of virus replication after vaccination. In accordance with the results from phase 1 clinical trials, the neutralizing antibody titers against DENV-1 and DENV-2 were higher than against DENV-3 and DENV-4 after administration of the first dose. On day 28, 94 % of the vaccinees were found to be seropositive for DENV-1, DENV-2, or DENV-3. Administration of the second dose resulted in increased GMT and seropositivity rates for DENV-4 (58.6 % after first dose to 87.7 % after second dose). The seropositivity rates for any of the DENV serotypes among dengue seropositive individuals increased from 91.3 %–99.1 % after the first dose to 96.5 %–100 % after the second dose, but there was no effect on the GMT of neutralizing antibodies [104]. Another round of phase 2 clinical trials whereby the TDV-2 dose was reduced to 5 × 10^3^ PFU was carried out to improve the immune responses of TDV-1, TDV-3, and TDV-4. The new vaccine formulation elicited more balanced immune responses, but much lower anti-DENV-2 neutralizing antibody titer was elicited when compared to the previous formulations, especially in dengue-naïve individuals. However, the amount of anti-DENV-4 neutralizing antibodies was still inadequate [105]. Apart from that, another round of large-scale phase 2 randomized, double-blind, placebo-controlled clinical trials was carried out to evaluate the safety and immunogenicity of three different vaccination schedules, consisting of administration of a single primary dose regimen at 0 month, one primary dose regimen at 0 month and one booster dose at 12 months, as well as two primary dose regimens at 0 and 3 months (NCT02302066). The vaccine formulation consisted of 2.5 × 10^4^ PFU of TDV-1, 6.3 × 10^3^ PFU of TDV-2, 3.2 × 10^4^ PFU of TDV-3, and 4 × 10^5^ PFU of TDV-4 [61]. All serotype-specific neutralizing antibodies were higher than the baseline at 6 months, 18 months, and 48 months. The vaccine formulation elicited neutralizing antibodies against all four DENV serotypes in vaccinees, with the highest immune response observed against DENV-2 and the lowest against DENV-4. The two primary dose regimens increased seropositivity rates against all four DENV serotypes and induced slightly higher long-lasting neutralizing antibody titers against DENV-1, DENV-3, and DENV-4. Although the 1-year booster dose increased the neutralizing antibody titers for a short period of time, especially in dengue-naïve individuals, the seropositivity rates and antibody concentrations were still similar to the two primary dose regimens at 48 months. These results suggested that the two dose schedules were more effective than the one dose schedule in increasing seropositivity rates in dengue-naïve individuals [[61], [62], [63]].

In the phase 3 randomized, double-blind, placebo-controlled clinical trials, the efficacy of TAK-003 was further evaluated in healthy children aged between 4 and 16 years old in eight dengue-endemic countries (NCT02747927). Two vaccine or placebo doses were administered with a 3 month gap and each vaccine dose consisted of 2.5 × 10^3^ PFU of TDV-1, 1 × 10^4^ PFU of TDV-2, 4 × 10^4^ PFU of TDV-3, and 1.2 × 10^5^ PFU of TDV-4. The follow-up post-second vaccination data after 12 months revealed that the overall vaccine efficacy was 80.9 % and the efficacy against dengue-related hospitalizations were 95.4 %. The vaccine efficacy varied for all four DENV serotypes, with 73.7 %, 97.7 %, and 62.6 % being reported against DENV-1, DENV-2, and DENV-3, respectively. Due to the limited number of DENV-4 cases, the vaccine efficacy against DENV-4 remained inconclusive [29,30]. The vaccine efficacy was found to be similar for all ages (72.8 %–83.3 %). The vaccine efficacies were determined to be 74.9 % for seronegative individuals and 82.2 % for seropositive individuals. Additionally, the vaccine efficacies for seropositives were 79.8 % and 96.5 % against DENV-1 and DENV-2, respectively. The vaccine efficacies for seronegatives were 67.2 % and 100 % against DENV-1 and DENV-2, respectively. Due to the limited number of confirmed DENV-3 and DENV-4 cases, the vaccine efficacies against DENV-3 and DENV-4 remained inconclusive, although no vaccine efficacy was suggested against DENV-3 [29]. On the other hand, the follow-up post-second vaccination data after 18 months reported an overall vaccine efficacy of 80.2 %. The overall vaccine efficacy for the timeframe between 12 and 18 months was determined to be 73.3 %. In addition, the vaccine efficacies for seropositive and seronegative individuals were determined to be 76.1 % and 66.2 %, respectively. For dengue haemorrhagic fever and dengue-related hospitalizations, the vaccine efficacies were determined to be 85.9 % and 90.4 %, respectively. The vaccine efficacies also varied among all the four DENV serotypes, with 69.8 %, 95.1 %, 48.9 %, and 51 % being reported against DENV-1 to -4, respectively. However, there was no data on vaccine efficacy against DENV-3 in seronegative individuals. Furthermore, TAK-003 also led to increased hospitalizations among dengue-naïve individuals due to DENV-3, but this data remained statistically inconclusive [30]. Next, the 24 months follow-up data reported an overall vaccine efficacy of 72.7 %. The vaccine efficacies for seropositive and seronegative individuals were determined to be 74.8 % and 67 %, respectively. The vaccine efficacies also varied among all four DENV serotypes, with 69 %, 90.8 %, and 51.4 % being reported against DENV-1 to -3, respectively, but efficacy against DENV-4 remained inconclusive. However, in the second year after vaccination, the vaccine efficacy dropped to 56.2 % and it was suggested that this could be due to a change in the predominant DENV serotype [64]. The 36 months follow-up data revealed the overall vaccine efficacy of TAK-003 to be 62 %. The vaccine efficacies for seropositive and seronegative individuals were determined to be 65 % and 54.3 %, respectively. Similarly, there was still a lack of vaccine efficacy against DENV-3 in dengue-naïve individuals. TAK-003 also led to increased hospitalization rates among dengue-naïve individuals due to DENV-3. Future follow-up studies should further investigate these findings as it could potentially indicate ADE in DENV-3-exposed seronegative individuals [56].

As of 2022, TAK-003 (currently known as Qdenga) was approved by the European Medicines Agency (EMA) for use in individuals >4 years old. It is administered subcutaneously with two doses given within an interval of 3 months. The Qdenga vaccine has been found to be well-tolerated and not linked to any serious adverse events. However, it is not recommended for use in pregnant and breastfeeding women as well as immunocompromised individuals [58]. Its efficacy was mainly studied in the Tetravalent Immunization against Dengue Efficacy Study (TIDES) trial, which involved 20,000 children and adolescents from eight countries in Asia and Latin America. In the first year post-vaccination, a vaccine efficacy of 80 % was observed against virologically confirmed dengue fever (VCD) and it was 95 % successful in preventing VCD-related hospitalizations. Its efficacy against VCD was the highest against DENV-2 at 98 % and the lowest against DENV-3 at 63 %. As the number of DENV-4 cases were limited in number, its efficacy against DENV-4 could not be determined at that time-point. At 4–5 years post-vaccination, the cumulative vaccine efficacy in preventing VCD dropped to 59 % and it was 84 % successful in preventing VCD-related hospitalizations. Its efficacy against VCD ranged between 43 and 82 % depending on the DENV serotypes. The vaccine efficacy was found to be higher in dengue seropositive individuals at 63 % when compared to dengue naïve individuals at 50 %. However, during the third year after administration of the second dose of the vaccine in dengue naïve individuals, large numbers of these vaccine recipients were hospitalized due to DENV-3. This indicated that the vaccine was not able to provide protective effect against VCD caused by DENV-3 in dengue naïve individuals, thereby indicating the need for booster doses [29,56]. Qdenga induced varying antibody responses against all four DENV serotypes, with the highest for DENV-2. However, in individuals >16 years old, no vaccine efficacy studies have been carried out. On the other hand, no vaccine studies have been carried out at all for individuals >60 years old [57].

#### 4.1.3 TV-003/TV-005/Butantan-DV vaccine

This tetravalent live-attenuated dengue vaccine was developed via the creation of a series of attenuated DENV with introductions of nucleotide deletions in the 3’-UTR and mutations in the NS proteins (Fig. 4) [65]. A total of six monovalent dengue vaccine candidates were then evaluated in mice and non-human primates with four covering all four DENV serotypes being selected for the final tetravalent vaccine formulation [66]. These candidates are rDEN1Δ30, rDEN2/4Δ30, rDEN3–3’D4Δ30, rDEN3Δ30/31, rDEN4Δ30, and rDEN4Δ30–200,201 [[106], [107], [108], [109], [110], [111], [112]]. Both rDEN1Δ30 and rDEN4Δ30 were constructed following a 30-nucleotide deletion (Δ30) in the 3’-UTR regions of DENV-1 and DENV-4 RNA genomes, respectively. rDEN3Δ30/31 included an additional 31-nucleotide deletion at 55 nucleotides upstream of the Δ30 mutation. rDEN2/4Δ30 was a chimeric virus obtained by replacing the prM and E genes of rDEN4Δ30 with those of DENV-2. rDEN3–3’D4Δ30 was also a chimeric virus obtained by substituting the whole 3’-UTR region of DENV-3 with the 3’-UTR region of rDEN4Δ30. Lastly, rDEN4Δ30–200,201 was created based on rDEN4Δ30 and it contained alanine substitutions at amino acid positions 200 and 201 of the NS5 protein. It was revealed in a previous study that these attenuated DENV could not be transmitted by *Aedes* mosquitoes [113].

In a randomized, double-blind phase 1 clinical trial, four different tetravalent formulations (TV001–004) from the six monovalent live-attenuated vaccine candidates were evaluated in dengue-naïve adults (NCT01072786) [66]. No difference in adverse events was observed between vaccine and placebo recipients. Most of the vaccinees reported only mild rash symptoms. Among the four vaccine formulations, it was revealed that TV-003 induced the most balanced antibody responses against all four DENV serotypes. Additionally, 97 % of the vaccinees were observed to develop trivalent neutralizing antibody responses after administration of a single dose of TV-003. It was also found that TV-003 induced higher neutralizing antibody titers against DENV-2, -3, and -4, indicating higher frequency of tetravalent responses, and slightly higher DENV-3 viremia in seropositive individuals. Furthermore, TV-003 was also able to elicit complete protection against DENV-2 in the human challenge model [114,115]. Next, administration of the TV-003 booster dose at 6 months or 12 months after the first dose were evaluated in dengue-naïve individuals to determine if the booster dose could increase neutralizing antibody titers (NCT01072786, NCT01782300). No severe adverse events were reported among the vaccinees for both trials. However, it was reported that 63 % of vaccinees from the 12 months booster trial experienced mild rash which lasted for an average of 7.7 days. The TV-003 booster dose administered at 6 months was found to increase seroconversion rates of DENV-2 from 76 % to 94 %. Data from both trials revealed that the TV-003 booster dose administered at 6 and 12 months could not increase the mean peak neutralizing antibody titers against any of the four DENV serotypes. Furthermore, it was found that the sterilizing immunity induced by the first vaccine dose could neutralize the booster vaccine dose, which indicated that the TV-003 booster dose was unnecessary with almost no benefits [67,116]. In two other clinical trials, TV-005 consisting of a 10-fold increase in the DENV-2 dose was evaluated (NCT01072786, NCT01436422). No severe adverse events were recorded and there were no significant differences in the adverse events between TV-003 and TV-005. It was observed that TV-005 increased the frequency of seroconversion and overall antibody titer against DENV-2, while maintaining its immunogenicity against the other DENV serotypes. Administration of a single dose of TV-005 elicited a tetravalent response in 90 % of the vaccinees, compared to 76 % of the TV-003 vaccinees after 3 months of vaccination [67].

The licensing of TV-003 by the Butantan Institute resulted in Butantan-DV, which is analogous to TV-003. Butantan-DV was then evaluated in phase 2 clinical trials involving both dengue-naïve and seropositive individuals (NCT01696422). The dosing regimen consisted of a single Butantan-DV dose, followed by a booster dose after 6 months. Similar to phase 1 clinical trials, most of the vaccinees (88 %–92 %) only experienced a mild rash. It was observed that there were no significant differences in the adverse events and post-vaccination viremia between dengue-naïve and seropositive individuals. The seroconversion rates at 91 days after administration of the first dose were observed to be 94 %, 82 %, 82 %, and 88 % for DENV-1 to -4, respectively. However, the seroconversion rates in dengue-naïve individuals were much higher for DENV-2 at 92 % and DENV-4 at 89 %, when compared to seropositive individuals at 78 % for DENV-2 and 77 % for DENV-4. Apart from that, the GMT of neutralizing antibodies were much higher in seropositive individuals for DENV-1 to -3, but not for DENV-4. Interestingly, administration of the booster dose did not improve the GMT of neutralizing antibodies and seroconversion rates. This was consistent with earlier observations whereby a single TV-003 dose was sufficient to induce immune protection against dengue [46,67,68].

There is an ongoing phase 3 clinical trial for Butantan-DV in Brazil involving 16,235 individuals (NCT02406729). It was initiated in 2016 and included both dengue-naïve and seropositive individuals aged between 2 and 59 years old. A single dose of the Butantan-DV vaccine was administered and follow-up studies were carried out to check for any adverse effect caused by any of the four DENV serotypes. The overall 2-year vaccine efficacy was determined to be 79.6 %. A higher vaccine efficacy of 89.2 % was attained in seropositive individuals whereas a lower vaccine efficacy of 73.6 % was recorded for dengue-naïve individuals. Additionally, the vaccine efficacies against DENV-1 and DENV-2 were determined to be 89.5 % and 69.6 %, respectively. Since DENV-3 and DENV-4 circulations were low during the course of the clinical trial, no data for vaccine efficacy was recorded. Apart from that, it was also revealed that seropositive individuals had a higher vaccine efficacy against DENV-1 at 96.8 % than dengue-naïve individuals at 85.5 %. Similar findings were reported for DENV-2 with a higher vaccine efficacy at 83.6 % for seropositive individuals and 57.9 % for dengue-naïve individuals. The current trial will continue to include a sufficient number of patients infected with DENV-3 and DENV-4 to determine the vaccine's performance against these two DENV serotypes [31,32]. Table 2 provides a comparison of the vaccine efficacies between CYD-TDV, TAK-003 and TV-003/TV-005 vaccines.

**Table 2:** Comparison of the vaccine efficacies between CYD-TDV, TAK-003/Qdenga, and TV-003/TV-005 vaccines.

Vaccine	Age group	Manufacturer	Vaccine efficacy (%)								References
			Overall	Seronegatives	Seropositives	DENV-1	DENV-2	DENV-3	DENV-4	Booster dose	
CYD-TDV/Dengvaxia®	2–17 years	Sanofi Pasteur	60	43.2	83.7	51	34	75	77	59 % (6 months post-third dose)	[[26], [27], [28]]
TAK-003/Qdenga⁎	4-16 years	Takeda	73.3	66.2	76.1	69.8	95.1	48.9	51	80.9 % (12 months post-second dose) 72.7 % (24 months post-second dose	[29,30]
TV-003/TV-005/Butantan-DV⁎⁎	2-59 years	Butantan Institute	79.6	73.6	89.2	89.5	69.6	N/D	N/D	N/D	[31,32]

N/D = Not determined as the results were inconclusive due to insufficient DENV-3 and DENV-4 cases.

⁎ Data from the TIDES trial.

⁎⁎ Data from an ongoing phase 3 clinical trial in Brazil.

### 4.2 Inactivated vaccines

Inactivated vaccines derived from viruses could provide immune protection against the live pathogen [117]. Inactivated vaccines generate immunity by utilizing antigens from the E, prM/M, C, and NS1 proteins of DENV, although composite vaccines provide better protection when compared to single-type vaccines. Additionally, inactivated vaccines are generally safer than live-attenuated vaccines as the immunological balance is well-regulated and there is no risk of virus reactivation [5]. Apart from that, inactivated vaccines could also avoid viral interference between live viruses in tetravalent formulation. However, the development of inactivated vaccines is limited by low immunogenicity [25]. An example of an inactivated DENV vaccine is the TDENV-PIV vaccine candidate developed by GlaxoSmithKline (GSK) and Walter Reed Army Institute of Research (WRAIR) (Table 1). It consists of all four DENV serotypes which were chemically inactivated using formalin to inhibit DENV infectivity, while maintaining their structures and antigenicity [71]. The monovalent DENV-1 PIV vaccine candidate which was evaluated in phase 1 clinical trial revealed that it has a good safety profile with mild adverse events such as minimal injection site reactions. Additionally, it was found that DENV-1 PIV generated seroconversion in all the study participants [72]. Despite its good safety profile, inactivated DENV vaccines have lower immunogenicity than live-attenuated vaccines. This could be overcome by co-administrating an adjuvant to increase the immune response. Two phase 1 clinical trials were carried out to determine the safety and efficacy of TDENV-PIV with different adjuvants such as aluminum hydroxide, AS03B, and AS01E. It was shown that these formulations were well-tolerated by all vaccinees and it induced a balanced immune response against all four DENV serotypes. Administrations of the TDENV-PIV vaccine with adjuvants AS03B and AS01E were found to induce the highest mean antibody titers [69,70]. Currently, the most effective administration schedule for TDENV-PIV with AS03B adjuvant is being evaluated in a phase 2 clinical trial [82,118]. Participants administered with the TDENV-PIV vaccine with AS03B adjuvant reported adverse events such as fatigue, gastrointestinal symptoms, headache, joint pain, muscle aches, and fever within 7 days after administration. Additionally, participants administered with three dose regimens also reported a high frequency of adverse events within 28 days after administration as compared to those administered with two dose regimens. However, no serious adverse events and potential immune-mediated diseases were reported, thus confirming the safety of the vaccine [73].

### 4.3 DNA vaccines

The development of DNA vaccines involves the incorporation of genes encoding specific antigens into a plasmid. Since the E protein anchored to the prM protein is responsible for mediating DENV-host cell interactions, it is the primary target for induction of neutralizing antibodies. This type of vaccine could induce both innate and adaptive immunity via *in vivo* injection of the recombinant plasmid expressing the respective antigens [119]. D1ME100 (otherwise known as TVDV or Vaxfectin®) is a DNA vaccine consisting of four plasmids carrying genes encoding for the E and prM proteins from each DENV serotype (Table 1). It was clinically tested on *Aotus nancymaae* monkeys and humans, and was proven to be safe and well-tolerated in the initial immunization phase. However, in participants who received high-dose immunization, weak immunogenicity was observed. On the other hand, in participants who received low-dose immunization, there was no neutralizing antibody response being detected [[74], [75], [76]]. This vaccine posed some concerns due to the DENV-specific IFN T-cell response generated in approximately 79 % of the high dosage vaccinees. Although this vaccine lacks immunogenicity, it has various advantages including good stability, ease of manufacture, good scalability, and low cost of production [74,77]. To address the drawbacks of this form of vaccine, modification of plasmid with more efficient promoters, alternative delivery approaches, multiple dosage, and addition of adjuvants could be included as possible solutions [5].

### 4.4 Recombinant subunit vaccines

This type of vaccine consists of utilizing antigenic proteins produced by prokaryotic or eukaryotic cells to generate long term immune protection [120]. The most common DENV recombinant subunit vaccine candidates are the shortened or E protein variants, which relied on the generation of neutralizing antibodies to prevent DENV infection of host cells. Although recombinant DENV proteins could be easily expressed in *Escherichia coli*, there are still concerns such as protein misfolding and exposure to endotoxins [83]. Recombinant subunit vaccines could generate steady immune responses against all four DENV serotypes as opposed to that of live-attenuated vaccines, thus reducing the probability of ADE [121]. A promising recombinant subunit vaccine candidate which has completed phase 1 clinical trials is V180, which is a recombinant DENV E vaccine developed by Merck (Table 1) [122]. In a preclinical study, V180 and ISCOMATRIX adjuvant were administered in mice and monkeys to evaluate their efficacy in providing protection against viremia and generating strong neutralizing antibodies against all four DENV serotypes [78,79]. In a phase 1 clinical trial, flavivirus-naïve adult participants were administered with V180 and the ISCOMATRIX adjuvant. All the vaccine formulations were well-tolerated by the study participants and strong immunity was observed among the study participants. Overall, the vaccine formulation with ISCOMATRIX adjuvants was found to be more immunogenic than vaccine formulations with aluminum adjuvants and without adjuvants [[80], [81], [82]].

### 4.5 Peptide-based vaccines

Synthetic peptide vaccines are made up of selected epitopes of the antigenic protein of interest for the induction of highly specific or targeted immune responses. This could prevent the possible risks associated with vaccines consisting of whole live pathogens such as reversion to virulence. In comparison to conventional vaccines, peptide-based vaccines have various advantages such as high reproducibility, cost effective, and fast synthesis. However, although it has a better safety profile as opposed to that of live-attenuated vaccines, synthetic peptide vaccines are known to be low in immunogenicity. Furthermore, peptides are also known to be unstable *in vivo* due to their susceptibility to enzymatic degradation [[123], [124], [125]]. Several studies have designed epitope-based peptide vaccines against DENV. A study by Gupta & Kumar (2022) designed an epitope-based peptide vaccine based on the DENV E protein using reverse vaccinology. The T-cell epitopes located in the DENV E protein were screened and chosen based on assessments of their immunogenicity, allergenicity, toxicity, and antigenic reactivity. They discovered three potent T-cell epitopes, IVQPENLEY, ILIGVVITW, and DTAWDFGSL, which were able to bind to the HLA-B*35:01, HLA-B*58:01, and HLA-A*26:01 alleles respectively. However, the predicted T-cell epitopes have yet to be validated by *in vitro* experiments [126]. In another study, an epitope-based peptide vaccine was developed based on the RNA-dependent RNA polymerase (RdRp) of DENV-2. The epitope, MGKREKKLGEFGKAKG, was revealed to be non-allergenic, non-toxic, and did not share any homology with the human genome. It was also shown to be able to form a stable bond with the receptors of dendritic cells with a binding free energy value of −474.4 kcal/mol. The authors hypothesized that the vaccine candidate would be structurally stable and able to generate good immune responses against DENV-2 infections. However, the predicted epitope has yet to be validated via *in vitro* experiments [127].

At present, only one peptide-based vaccine against dengue, a gold nanoparticle-based, multivalent, synthetic peptide dengue vaccine candidate (PepGNP-Dengue) has been evaluated in a phase 1 double-blind, randomized, vehicle-controlled clinical trial (NCT04935801) (Table 1). This vaccine was designed to provide CD8^+^ T-cell immunity, without eliciting anti-DENV antibodies. In this study, healthy, flavivirus-naïve individuals aged between 18 and 45 years old were randomly assigned to receive two low or high doses of PepGNP-Dengue or vehicle-GNP (without peptides) 21 days apart. Only mild adverse events such as pain at the injection site and transient discoloration of the skin were reported. It was also shown that PepGNP-Dengue did not elicit anti-DENV antibodies as expected. In the low dose PepGNP-Dengue group, significant increases in the CD8^+^ T cells and dengue dextramer+ memory cell subsets were observed, but not in the high dose PepGNP-Dengue or vehicle-GNP groups. The authors hypothesized that the weaker immune response observed in the high dose PepGNP-Dengue group could be attributed to the increased duration of vaccine retention at the injection site [84]. It was previously found that antigen persistence at the site of vaccination induced sequestration of specific CD8+ T-cells [128].

Apart from that, there are several experimental peptide vaccine candidates which have been evaluated in *in vitro* and *in vivo* experiments. A study by Li et al. (2011) designed a multi-epitope peptide vaccine containing B- and T-cell epitopes of domain II of the DENV-2 E protein. The two epitope peptides, designated A1 and A2, coupled to the keyhole limpet hemocyanin (KLH) carrier protein exhibited good specificity and biological activity. These two epitope peptides together with the pan-HLA-DR-binding epitope (PADRE) peptide formed the multi-epitope peptide vaccine, designated as P1. Their findings revealed that the P1 vaccine was highly immunogenic and able to induce both cellular and humoral immune responses. It was also observed that a high level of antibody titer was elicited in C57BL/6j mice inoculated with the P1 vaccine. Additionally, DENV-2-infected C57BL/6j mice vaccinated with the P1 vaccine demonstrated lesser virus replication than the control group [129]. In another study by Chan et al. (2020), four multi-epitope peptides, P1-P4 were developed by linking a universal T-helper epitope such as PADRE or TpD to highly conserved B- and CD8^+^ T-cell epitopes across all four DENV serotypes. The multi-epitope peptides were then conjugated to polystyrene nanoparticles to form four nanovaccines, NP1-NP4. The findings from the mice immunization experiments revealed that NP1-NP4 induced significantly higher IgG and neutralizing antibody titers when compared to that of naked P1-P4. Among the four peptide nanovaccines, it was observed that NP3 was able to induce significant levels of IFN-γ and neutralizing antibodies against all four DENV serotypes [130].

### 4.6 mRNA vaccines

The mRNA vaccine technology has been utilized in the development of vaccines for many viral infectious diseases such as Zika virus [131], Ebola virus [132], human immunodeficiency virus (HIV) [133], and influenza virus [134]. In comparison to DNA vaccines, mRNA vaccines cannot integrate into the host genome. This avoids any potential risk of insertional mutagenesis and potential oncogenesis. This type of vaccine generally consists of a 5′ cap at the 5’UTR, genes encoding for one or more antigens, and a poly(A) tail at the 3’UTR [86]. Wollner et al. (2021) developed an mRNA dengue vaccine by transcribing the DENV-1 prM/E proteins into a modified mRNA vaccine candidate using pseudo-uridine, and then packing it into lipid nanoparticles (LNP) (Table 1). The mRNA-LNP complex was administered via intramuscular injections into AG129 mice. Following endocytosis into muscle cells, the LNP was degraded, leaving behind the mRNA for translation giving rise to the viral prM/E proteins. These viral proteins then exerted DENV-1-specific humoral and cellular immune responses in the AG129 mice [85]. Another study by Zhang et al. (2020) targeted prME, E80, and NS1 proteins of DENV-2 using the same mRNA vaccine technology. This vaccine candidate was able to induce the production of neutralizing antibodies against DENV-2 and generated T cell immune responses in the BALB/c mice [86]. A study by Mukhtar et al. (2022) developed a tetravalent modified nucleotide mRNA vaccine using B cell epitopes, CD8^+^ T cell epitopes, and CD4^+^ T cell epitopes present in DENV NS1, prM and EIII sequences. The B and T cell epitopes were predicted based on the assessment of the antigenicity, allergenicity, immunogenicity, and toxicity profiles [135]. He et al. (2022) constructed another modified mRNA vaccine coated with LNP, which encodes for DENV-1 and DENV-4 EIII sequences as well as DENV-2 and DENV-3 NS1 sequences. This vaccine was able to induce good antiviral immune responses and high neutralizing antibody titers to block DENV-1 to -4 infections *in vitro* without significant ADE [136].

## 5 Challenges in the development of dengue vaccines

Despite the availability of the Dengvaxia® vaccine, its ability to provide long-lasting, balanced immune protection against all four DENV serotypes remained challenging. Moreover, it can only be used to vaccinate seropositive children. The development of a dengue vaccine is affected by various factors such as DENV transmission and epidemiology, disease pathogenesis and immunological processes involved, as well as the characteristics of DENV. In dengue endemic regions, a protective antibody response against the respective infection is obtained through the recurrence of viral exposure. After infection with any of the four DENV serotypes, the patient could be re-infected with any of the other DENV serotypes. Therefore, if there is a vaccine which could provide cross-protection between all four DENV serotypes, there would no longer be a need to further develop a dengue vaccine. An ideal dengue vaccine must be able to elicit a multitype immune response similar to that of individuals living in dengue-endemic regions with persistent exposure to DENV as well as being asymptomatic or symptomatic upon DENV re-infection. However, significant challenges such as ADE, cross-reactivity with other flaviviruses, and the multiple DENV serotypes limit the development of an efficient dengue vaccine [5,81].

The main challenge in dengue vaccine development is the complexity of the virus itself. Infection with a single serotype generates life-long immune protection against that specific serotype and short-lived immune protection against the other three heterologous serotypes [12]. This could lead to severe dengue in subsequent infections, which could potentially be contributed by ADE. This proves to be a challenge for dengue vaccine development as it necessitates a tetravalent vaccine with balanced, long-term protection against all four DENV serotypes simultaneously [137]. Next, the immunological variability of individuals due to factors such as age, prior exposure to DENV, and co-infections also poses a great challenge to dengue vaccine development. This was a significant issue encountered with the Dengvaxia® vaccine as it enhanced the risk of hospitalization for seronegative vaccinees [50]. Furthermore, the lack of a clear cut-off point for protective immunity presents another challenge to dengue vaccine development. This cut-off point refers to the correlates of protective immunity such as antibody titers and virological titers. The endpoints used for the evaluation of vaccine efficacy in clinical trials can be affected by factors such as age of the participants, immune status of the participants, and the dengue transmission levels in the community [137].

The lack of suitable animal models which exhibit similar symptoms attributed by dengue as what is observed in humans is another major problem which poses a challenge to the development of dengue vaccines. Currently, the AG129 mice strain which is deficient in IFN α/β/γ receptors is the most suitable mouse model for the study of dengue replication *in vivo* [138]. It was revealed that this mouse strain could induce DENV-specific antibodies and protect against both heterologous and homologous virus challenges [139]. Apart from that, the development of humanized mice through engraftment of human hematopoietic progenitors in immunocompromised mice strains such as BALB/c-*Rag2*^*null*^ *ILR2rγ*^*null*^ (BRG) and NOD/*scid/ILR2rγ*^*null*^ (NSG) could be beneficial for future dengue vaccine development and pathogenesis studies [140,141]. These mice models contain human immune cells such as monocytes, dendritic cells, B- and T-cells which serve as targets for dengue infections. Humanized mice models could be used for preliminary observations of human immune responses upon administration of the new vaccine candidates. Furthermore, the use of humanized mice models could also eliminate the need for large animal models such as non-human primates which are limited by concerns such as ethical issues, high cost, and requirements for special housing conditions. However, currently, humanized mice models still do not possess an established human immune system and might not provide reliable, accurate results [25].

## 6 Dengue human infection model (DHIM)

Apart from animal models, another potent tool for evaluations of dengue vaccines is the dengue human infection model (DHIM). DHIM provides important information such as the safety and immunogenicity of the vaccine formulations via testing in a small group of individuals. The first type of DHIM was developed by the United States National Institutes of Health (NIH), whereby a modified version of the DENV-2 strain, DEN2Δ30 which caused mild clinical symptoms and induced viremia in all 30 participants (infectious model). The second type of DHIM was developed by WRAIR to determine the DENV strain which caused symptomatic dengue fever (infection and disease model) [142,143]. Therefore, pre-existing dengue infections could be established using a DHIM to mirror evaluations in dengue-endemic regions. DHIM could also provide parameters such as the type of T-cell immune responses and neutralizing antibody titers which correspond to immune protection. The main objective of DHIM is to select the most suitable dengue vaccine candidate or virus challenge strain. It differs from phase 1 clinical trial because it must be performed in an early phase, strictly controlled clinical study [25].

DHIM could support various components of the vaccine clinical development. Vaccine developers and researchers could evaluate the safety and efficacy of the vaccine candidates in different vaccinees. DHIM could allow vaccine developers and researchers to create dengue primed individuals for preliminary safety testing of vaccine candidates in strictly controlled and low risk environments. It is also possible to evaluate the impact of dengue priming on safety and the priming of different DENV types using DHIM. DHIM could also aid in decisions concerning early vaccine formulation such as antigen concentration, antigen selection, dosage, and adjuvant selection. Therefore, DHIM would be valuable for vaccine development efforts due to its high predictive value [143].

## 7 Conclusion

The first licensed dengue vaccine, CYD-TDV (Dengvaxia®) is limited in use due to an elevated risk of severe dengue infections among dengue-naïve vaccinees (particularly in children below 9 years of age) and its low efficacy against DENV-2. Although the Qdenga vaccine showed some promising results, its efficacy against DENV-3 was significantly lower than that of DENV-1 and DENV-2. Therefore, the search for a more efficient tetravalent dengue vaccine candidate is still on-going. Most of the vaccine candidates are still undergoing preclinical or clinical trials to assess their safety and efficacy in humans on a longer term. Furthermore, the development of dengue vaccines faced various challenges such as the lack of suitable animal models, immunological variability of individuals, the lack of a clear cut-off point for protective immunity, and the need for long term protection against all four DENV serotypes. At present, the development of a dengue vaccine that could confer balanced immune protection against all four DENV serotypes remains a challenge.

## Funding

Not applicable.

## CRediT authorship contribution statement

**Michelle Felicia Lee:** Writing – review & editing, Writing – original draft, Software, Methodology, Conceptualization. **Chiau Ming Long:** Writing – review & editing, Validation, Supervision, Software. **Chit Laa Poh:** Writing – review & editing, Writing – original draft, Validation, Supervision, Methodology, Formal analysis, Conceptualization.

## Declaration of competing interest

The authors declare that they have no known competing financial interests or personal relationships that could have appeared to influence the work reported in this paper.

## Data Availability

No data was used for the research described in the article.
